# Risk Factors for Asymptomatic Ventricular Dysfunction in Rheumatoid Arthritis Patients

**DOI:** 10.1155/2013/635439

**Published:** 2013-12-03

**Authors:** Carlos Garza-García, Sánchez-Santillán Rocío, Arturo Orea-Tejeda, Lilia Castillo-Martínez, Canseco Eduardo, José Luis López-Campos, Candace Keirns-Davis

**Affiliations:** ^1^Internal Medicine Department, Instituto Nacional de Ciencias Médicas y Nutrición “Salvador Zubirán”, 03100 Mexico City, DF, Mexico; ^2^Heart Failure Clinic, Instituto Nacional de Ciencias Médicas y Nutrición “Salvador Zubirán”, 03100 Mexico City, DF, Mexico; ^3^Cardiology Department, Instituto Nacional de Ciencias Médicas y Nutrición “Salvador Zubirán”, 03100 Mexico City, DF, Mexico; ^4^Massachusetts General Hospital Interpreter Services, Boston, MA 02114, USA

## Abstract

*Objective*. The aim of the study was to describe echocardiographic abnormalities in patients with rheumatoid arthritis, concurrent systemic comorbidities, rheumatologic clinical activity, serologic markers of rheumatoid arthritis, and inflammatory activity. *Methods*. In an observational, cross-sectional study, rheumatoid arthritis outpatients were included (*n* = 105). Conventional transthoracic echocardiographic variables were compared between patients with arthritis and non-RA controls (*n* = 41). For rheumatoid arthritis patients, articular activity and rheumatologic and inflammatory markers were obtained. *Results*. Ventricular dysfunction was found in 54.3% of the population: systolic (18.1%), diastolic (32.4%), and/or right (24.8%), with lower ejection fraction (*P* < 0.0001). Pulmonary hypertension was found in 46.9%. Other echocardiographic findings included increased left atrial diameter (*P* = 0.01), aortic diameter (*P* = 0.01), ventricular septum (*P* = 0.01), left ventricular posterior wall (*P* = 0.013), and right ventricular (*P* = 0.01) and atrial diameters compared to control subjects. Rheumatoid factor and anti-CCP antibodies levels were significantly elevated in cases with ventricular dysfunction. Angina and myocardial infarction, diabetes, and dyslipidemia were the main risk factors for ventricular dysfunction. *Conclusions*. Ventricular dysfunction is common in rheumatoid arthritis and associated with longer disease duration and increased serologic markers of rheumatoid arthritis. Screening for cardiac abnormalities should be considered in this kind of patients.

## 1. Background

Rheumatoid arthritis (RA) is a chronic inflammatory disease [1–4], and even though its major characteristic is polyarticular affection it is associated with such extra-articular features as vasculitis, keratoconjunctivitis sicca, bronchiolitis obliterans, organizing pneumonia, portal fibrosis, secondary amyloid, and cryoglobulinemia [5]. It has also been associated with increased cardiovascular risk due to conduction and valve alterations, heart failure, and premature atherosclerosis [6–8].

Multiple studies have shown that accelerated and increased atherosclerosis in autoimmune diseases leads to ischemic coronary artery disease. The proposed mechanisms for this are a major systemic inflammatory state involving T cell lymphocytes, tumor necrosis factor alpha (TNF alpha), high density lipoprotein dysfunction, and treatment related hyperhomocysteinemia. All of these factors lead to thickening of arterial intima, myocardial dysfunction, and in some cases myocardial infarction and are responsible for up to 50% of deaths in this population [9].

RA has been linked to several structural heart abnormalities, including increased right ventricular filling pressure, left ventricular hypertrophy, pulmonary artery hypertension, and as much as twice the prevalence of heart failure [5, 8, 10]. However, many patients have been found to develop asymptomatic diastolic dysfunction demonstrated by echocardiographic studies [11, 12] and by cardiac magnetic resonance. Giles et al. found that mean LV mass was strikingly lower in RA compared to controls [13], so RA patients represent an important target population for early management in strategies to reduce progression to advanced heart failure.

Cardiovascular risk and RA disease activity assessment through clinical judgment and appropriate questionnaires in addition to inflammatory markers may provide adequate screening tools to predict heart disease in RA patients, even in asymptomatic stages, which eventually will lead to early, cost effective studies and treatment.

## 2. Methods

We performed an observational cross-sectional study among outpatients of the Internal Medicine and Cardiology Departments of the Instituto Nacional de Ciencias Médicas y Nutrición SZ. Subjects were included if they were at least 18 years old with a previous diagnosis of rheumatoid arthritis. Patients with suspected heart failure, cardiomyopathy, endocarditis or myocarditis, amyloidosis, chronic obstructive pulmonary disease, pulmonary hypertension, secondary Sjögren's disease, infiltrative diseases, severe anemia hematologic disorders, and severe kidney and/or hepatic disease were excluded. Control subjects were outpatients of the Internal Medicine Department with an echocardiogram study and without diagnosis of rheumatoid arthritis who were matched for age and sex with rheumatoid arthritis.

Selected patients completed a clinical history and physical examination, emphasizing current and former medications, cardiovascular diseases and other comorbidities, and symptoms and signs of RA activity within the last 7 days. If articular activity was found, an analog visual scale for pain and DAS28 questionnaire were applied to establish RA activity at the time.

All patients had transthoracic echocardiograms performed by a cardiologist blinded to the clinical evaluation and treatment received. Wide angle, two-dimensional echoes were videotaped on a 3/4 inch videocassette recorder with patients in left lateral recumbent position, using an HP Sonos ultrasound 5000 imaging system. Apical four chambers and apical two chambers views were selected for left ventricular long axis at end-diastole and at end-systole to calculate ejection fraction and stroke volume. Total peripheral resistances were estimated using standard formulas according to the recommendations of the American Society of Echocardiography.

Ventricular dysfunction was diagnosed following the echocardiography criteria of the American Heart Association, the American College of Cardiology, and the European Society of Cardiology. Diastolic dysfunction was considered to be present when the ejection fraction was more than 45% and the shortening fraction ≥28%, without severe segmental dyskinesia of the left ventricle and left atrial enlargement or increased thickness of the posterior wall, ventricular septum, and cardiac mass index.

The study was approved by the Institutional Ethics Committee of Biomedical Research in Humans of the INCMNSZ, and all participants gave written informed consent.

### 2.1. Statistical Analysis

Data are presented as mean ± standard deviation for continuous variables with similar distribution to the normal curve. In the case of categorical variables, data are presented as absolute and relative frequencies. Study population was classified in two groups: patients with RA and ventricular dysfunction and patients with RA without ventricular dysfunction. The groups were compared with a Student's *t*-test for independent samples or chi-square according to the type of variable. Echocardiographic findings of patients with RA were compared to those of a control group without RA. A *P* value <0.05 was considered statistically significant. All analyses were performed with commercially available software (SPSS 17.0 for Windows, SPSS, Inc., Chicago, IL, USA).

## 3. Results

In the present study 105 patients with RA were included; the female gender (79%) was predominant. Hypertension (51.4%), coronary artery disease (27.6%), and diabetes (17%) were the most frequent cardiovascular risk factors.

Fatigue (42.9%), shortness of breath (49.5%), and edema (39%) were the main symptoms reported potentially associated with heart failure. From the rheumatologic perspective, most patients were in functional categories 1 or 2, with no significant limitations of daily activities. According to the DAS questionnaire, most patients had high or intermediate disease activity at the time of evaluation, 30.5% high and 42.9% intermediate activity. Glucocorticoids, folic acid antagonist (methotrexate), and other disease modifying antirheumatic drugs (DMARD) were used in 26.7% of the patients.

Among the echocardiographic findings, patients with RA had lower ejection fractions, greater left atrial and aortic diameters, ventricular septum and left ventricular posterior wall thickness, and right ventricular and atrial diameters. Pulmonary systolic arterial pressure (PSAP) was also increased in comparison to control subjects (Table 1).

Echocardiographic abnormalities indicating ventricular dysfunction were found in 54.3% of the population, including left systolic (18.1%), diastolic (32.4%), and/or right (24.8%) ventricular dysfunction. It was also frequent to observe pulmonary hypertension (46.9%).

When patients with RA without ventricular dysfunction were compared to RA patients with ventricular dysfunction, the latter were older, but there were no differences between the groups with respect to the duration of rheumatologic disease. Angina and myocardial infarction, diabetes, and dyslipidemia were frequently found in the patients with ventricular dysfunction. However, serum cholesterol concentrations were lower in patients with ventricular dysfunction, although, LDL cholesterol was slightly higher. HDL concentrations were similar in both groups (Table 2).

With respect to rheumatoid factor and anti-CCP antibodies levels, these were also significantly elevated in cases with ventricular dysfunction, despite similar erythrocyte sedimentation rates in the two groups (Table 2).

The use of glucocorticoids, folic acid antagonist (methotrexate), or other DMARDs was not different across RA groups, while treatment with beta blockers, diuretics, digitalis, and nitrates was significantly more prevalent in those patients with echocardiographic anomalies (*P* < 0.05).

In the multivariable analysis by logistic regression, only diabetes and cardiovascular disease were independent risk factors for ventricular dysfunction with odds ratios of 14.7, *P* = 0.02 and 6.43, and *P* = 0.04, respectively.

## 4. Discussion

Heart disease in patients with rheumatoid arthritis remains one of the main causes of death in this population [8, 14, 15]. In the present study structural heart disease (biventricular abnormalities and pulmonary hypertension) was found in 53% of the patients in this study. These echocardiographic findings in a Mexican population with rheumatoid arthritis agree with those reported in patients from Italy, Poland and the United Kingdom [5, 16, 17]. Diastolic dysfunction was the most common functional alteration along with pulmonary artery hypertension as previously reported [5, 16]. Major variables associated with structural heart disease were increased such as age, cardiovascular disease, and diabetes mellitus as clinical entities. Among the laboratory parameters we found that rheumatoid factor and anti-CCP antibodies correlated with echocardiographic findings, as Vizzardi and Cavazzana described [16].

Another interesting finding was that treatment with DMARDS, folic acid antagonists, glucocorticoids, and folic acid supplements was not significantly different between RA patients with asymptomatic ventricular dysfunction and those without. To be sure, treatment with beta blockers, diuretics, digitalis, and nitrates was significantly more prevalent in those patients with echocardiographic anomalies (*P* < 0.05). Whether these drugs were capable of maintaining patients asymptomatic is not known.

Ventricular dysfunction and RA have a proinflammatory chronic state, with overactivity of cytokines as TNF-*α* widely accepted by its participation in ventricular dysfunction, inducing apoptosis in cardiomyocytes and negative ventricular inotropic effect, myocardial fibrosis, and ventricular remodeling [18, 19]. As well as the presence of atherosclerosis that is associated with the frequent chronic steroid therapy involving metabolic abnormalities, such as lipid, carbohydrates metabolism, and endothelial dysfunction [20].

As in other forms of cardiovascular disease, endothelial dysfunction plays a central role in functional disturbances that involve coronary, pulmonary, and systemic arteries reducing the tissue perfusion and their functionality. In this way, unexpectedly, neither the glucocorticoids, folic acid antagonist (methotrexate), nor DMARDs were different between the groups in the present study, but diabetes and coronary artery disease traditional risk factors were independently associated with ventricular dysfunction. These findings are different from those reported by other authors that mention that the risk of developing CHF in RA is twice the risk of developing CHF in persons without RA and is not explained by traditional cardiovascular risk factors and/or clinical ischemic heart disease [8].

We consider that RA patients with the clinical and serologic characteristics previously mentioned should be screened for ventricular dysfunction and pulmonary hypertension. Even in clinically asymptomatic patients these entities must be ruled out, since early intervention may prevent the development of clinically symptomatic heart failure and severe pulmonary hypertension, and in later stages interventions will not provide as good a prognosis.

## 5. Conclusion

The present study illustrates that some rheumatoid arthritis patients present structural heart abnormalities that will probably progress to heart failure. These abnormalities appear to be more common in patients with a history of coronary artery disease and diabetes, longer disease duration, and increased serologic markers of rheumatoid arthritis. Focused assessment directed toward heart failure screening based on clinical judgment and echocardiography in a population with the former characteristics offers an opportunity for appropriate and early management of heart disease.

## Figures and Tables

**Table 1:** Echocardiographic findings in RA patients compared with normal controls.

Variable	Patients with RA *n* = 105	Controls *n* = 41	*P*
Age (years)	62.4 ± 14.6	57.4 ± 13.9	0.09
Gender (male/female), *n* (%)	22 (21)/83 (79)	10 (24.4)/31 (75.6)	0.65
Shortening fraction (%)	35.8 ± 8.6	39.4 ± 11.8	0.06
Ejection fraction (%)	60.0 ± 12.1	67.7 ± 5.8	<0.0001
Left ventricular diastolic diameter (mm)	43.5 ± 6.4	41.3 ± 6.2	0.06
Left ventricular systolic diameter (mm)	28.2 ± 7.2	25.0 ± 5.7	0.013
Left atrial diameter (mm)	38.6 ± 6.3	32.5 ± 9.8	0.01
Aortic diameter (mm)	29.5 ± 3.6	26.1 ± 6.9	0.01
Ventricular septum (mm)	10.6 ± 1.8	9.3 ± 1.7	0.01
Left ventricular posterior wall (mm)	9.9 ± 1.7	9.1 ± 1.5	0.013
Right ventricular diastolic diameter (mm)	31.9 ± 6.7	22.2 ± 13.4	0.01
Pulmonary artery systolic pressure (mmHg)	41.9 ± 14.9	28.3 ± 8.6	0.01
Right atrium (mm)	36.0 ± 6.9	28.0 ± 13.3	0.002
E/A ratio	0.91 ± 0.31	0.80 ± 0.23	0.19
E wave deceleration time TDE (mm/s)	215.1 ± 76.1	231.0 ± 69.0	0.29

**Table 2:** Comparison of clinical and biochemical characteristics between rheumatoid arthritis patients with and without ventricular dysfunction.

Variables	Ventricular dysfunction n = 57	No ventricular dysfunction n = 48	P
Age (years)	65.4 ± 14.1	58.8 ± 14.6	**0.02**
Habitual weight (kg)	65.1 ± 10.9	63.0 ± 11.2	0.36
Body mass index (kg/m^2^)	23.6 ± 9.1	24.0 ± 8.8	0.83
Rheumatoid arthritis evolution time	18.87 ± 12.54	15.09 ± 11.12	0.11
Cancer (%)	9.6	14	0.51
Coronary artery disease (%)	45.3	11.9	**<0.0001**
Obesity (%)	21.2	25	0.65
Diabetes (%)	26.4	9.3	**0.03**
Dyslipidemia (%)	11.5	32.6	**0.01**
Hypertension (%)	56.6	54.5	0.84
Nephropathy (%)	15.1	14	0.87
Albumin (mg/dL)	3.34 ± 0.67	3.41 ± 0.52	0.38
Hemoglobin (g/dL)	12.7 ± 2.1	13.1 ± 2.0	0.39
Hematocrit (%)	38.1 ± 6.1	38.9 ± 5.7	0.39
Total cholesterol (mg/dL)	173.0 ± 54.4	177.3 ± 37.0	**0.047**
c-LDL (mg/dL)	107.2 ± 43.0	101.0 ± 23.0	**0.005**
c-HDL (mg/dL)	42.6 ± 13.6	42.3 ± 13.2	0.78
Triglycerides (mg/dL)	129.2 ± 59.5	159.0 ± 87.7	**0.04**
DAS28 questionnaire	4.74 ± 1.13	4.53 ± 1.31	0.18
Rheumatoid factor UI/dL	96.5 (63.9–631.7)	37 (20.8–220.8)	**0.03**
Anti-CCP antibody	264 (23–136.5)	114 (18.9–784.3)	0.34
C-reactive protein	1.65 (0.89–2.85)	1.03 (0.57–4.08)	0.34
ESR (mm/hr)	29 (18–48.8)	33 (18.8–57)	0.5
Functional capacity			
Stage 1	18 (36)	24 (53.3)	
Stage 2	24 (48)	12 (26.7)	0.13
Stage 3	8 (16)	8 (17.8)	

Anti-CCP: anticyclic citrullinated peptide antibodies; ESR: erythrocyte sedimentation rate.

Data are expressed as %, mean ± standard deviation, or median (percentile 50–75).
